# Automated quantification of mesenteric hyperaemia in Crohn’s disease using time-of-flight magnetic resonance angiography

**DOI:** 10.1007/s00261-026-05384-6

**Published:** 2026-02-06

**Authors:** Iyad Naim, Caroline Hoad, Penny Gowland, Christopher Clarke, Rahul Munyal, Ana-Maria Darie, Sunil Samuel, Gordon W. Moran

**Affiliations:** 1 Sir Peter Mansfield Imaging Centre, University of Nottingham, Nottingham, United Kingdom; 2 Nottingham University Hospitals NHS Trust, Nottingham, United Kingdom

**Keywords:** Crohn’s disease, Comb sign, Mesentery, Magnetic resonance angiography, Hyperaemia, Vasa recta

## Abstract

**Background:**

Mesenteric hypervascularity (“comb sign”) reflects active inflammation in Crohn’s disease (CD) but is judged qualitatively on cross-sectional imaging. Despite its clinical relevance, no objective, quantitative MRI-based measures exist to assess this feature. This study aims to develop and evaluate an automated, contrast-free time-of-flight MR angiography (ToF-MRA) method for quantifying mesenteric vascularity in CD by comparing these measurements with healthy controls and assess the method’s repeatability and susceptibility to confounding by adiposity or prior bowel resection surgery.

**Methods:**

Twenty-three patients with active CD and seventeen healthy controls underwent abdominal ToF-MRA scans. A fully automated pipeline was developed to segment mesenteric vessels on 18 rotating maximum-intensity projections, skeletonised vessels, and count branching points to compute an Arborisation Index. Secondary analyses included a BMI-matched sub-cohort, a prior-resection comparison, and associations with BMI and visceral adipose tissue from Dixon MRI scans. Repeatability was assessed using repeated scans acquired during the same visit.

**Results:**

The Arborisation Index was higher in CD patients than in healthy controls (122.7 ± 37.1 vs. 98.4 ± 24.6; *p* = 0.02; d = 0.7) and this difference persisted in 10 BMI-matched pairs (145 ± 36 vs. 103 ± 24; *p* = 0.008; d = 1.4). Prior bowel resection (*n* = 11) did not influence the measure (*p* = 0.42). Short-term repeatability was good, with coefficients of variation of 8.9 ± 6% in CD and 8.5 ± 5% in controls. The Arborisation Index showed no significant correlation with BMI or visceral adipose volume.

**Conclusion:**

The Arborisation Index demonstrated significant group-level differences between CD patients and healthy controls, showed good short-term repeatability, and appears technically robust to adiposity and surgical history. Larger longitudinal studies are required to establish its biological meaning, clinical relevance, and responsiveness to inflammatory activity.

**Graphical Abstract:**

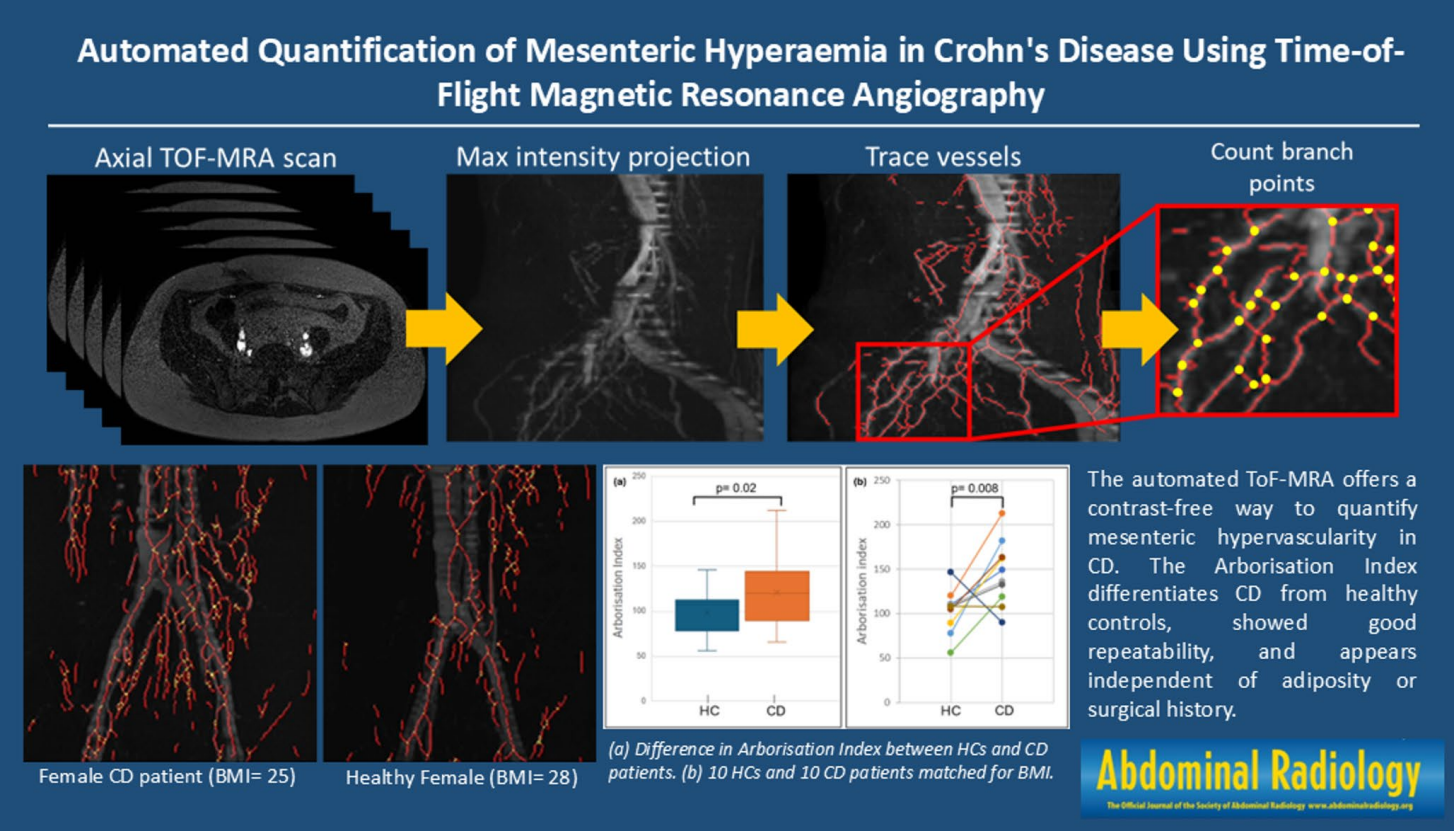

**Supplementary Information:**

The online version contains supplementary material available at 10.1007/s00261-026-05384-6.

## Introduction

Crohn’s disease (CD) is a chronic relapsing and remitting inflammatory bowel disorder, often associated with significant morbidity. Accurate assessment of disease activity is crucial for guiding therapeutic decisions and monitoring treatment response. Cross-sectional imaging plays a central role in this evaluation, and radiologists routinely assess both mural and extramural features to characterise inflammatory burden [1].

Mesenteric hypervascularity—the “comb sign”—is a well-recognized marker of CD on cross-sectional imaging, defined by dilated, tortuous, and widely spaced vasa recta along the mesenteric border of inflamed bowel. On contrast-enhanced CT, these engorged vasa recta branch in parallel and appear aligned like the teeth of a comb [2].

Historically, the comb sign has been described in contrast-enhanced computed tomography (CT) and magnetic resonance enterography (MRE) studies and used primarily as a qualitative indicator of active inflammation [2–4]. However, despite its clinical relevance, there has been no objective and reproducible MRI-based method to quantify the comb sign, limiting its use as a standardized biomarker in medical practice and clinical trials.

Previous studies have attempted to correlate subjective grading of the comb sign with CD activity using CT imaging. These studies demonstrated an association between the presence of mesenteric hypervascularity and endoscopic disease severity [5, 6]. However, reliance on contrast-enhanced imaging and subjective evaluation restricts the utility of this method for long-term disease monitoring, particularly given concerns about ionizing radiation exposure [7].

While Phase-Contrast Magnetic Resonance Angiography (PC-MRA) offers quantitative measurement of blood flow, its prolonged scanning time and limited imaging volume make it not the sequence of choice for routine clinical use on large areas like the abdominal cavity [8]. Similarly, Contrast-Enhanced MRA provides detailed inflow dynamics but with higher expense, potential contrast agents safety concerns [9], and limited spatial resolution due to rapid imaging requirements during contrast inflow [10].

Time of Flight Magnetic Resonance Angiography (ToF-MRA) is a non-contrast imaging technique that is used extensively across multiple clinical settings due to its ability to capture high-resolution images of blood flow. Unlike other MRA methods, ToF-MRA relies on the natural flow-related enhancement of moving blood spins, which appear significantly more hyperintense than stationary tissues when entering a magnetically labelled imaging slice. This feature makes ToF-MRA particularly effective for visualising the vascular structures of the brain, where it is routinely employed to assess cerebral aneurysms and occlusive vascular diseases [11, 12]. Beyond neurovascular applications, ToF-MRA has been adapted for peripheral artery disease evaluations, providing a safer alternative for patients at risk of nephrogenic systemic fibrosis from contrast agents [13].

By harnessing these inflow enhancement properties, ToF-MRA offers a distinct advantage in the detailed assessment of vessel morphology and has been progressively refined to improve its quantitative capabilities through software advancements and higher magnetic field strengths.

The primary aim was to quantify mesenteric hypervascularity on contrast free ToF-MRA using a fully automated algorithm that computes an Arborisation Index, an objective measure of abdominal vessel branching in Crohn’s disease.

## Methods

### Participant recruitment

This study was conducted in strict accordance with the principles of the Declaration of Helsinki. Ethical approval was obtained from the University ethics committee for recruiting healthy controls (ref: 197–1901 issued: 17/05/2019) and the NHS Yorkshire & The Humber - South Yorkshire Research Ethics Committee approval for recruiting CD patients with CD (ref: 19/YH/0337 issued: 26/11/2019). All participants provided written informed consent before participation.

We recruited adult patients (> 16 years) with active Crohn’s disease defined by sMaRIA [14] score ≥ 1 or C-reactive protein (CRP) ≥ 5 mg/L or faecal calprotectin (FCP) ≥ 50 µg/g. Surgical history, CRP and FCP results temporally closest to the ToF-MRA date were collected from hospital records. Global sMaRIA was scored on each participant’s from routine clinical MRE by two GI radiologists in consensus (CC, RM; >8 years’ experience). The values and dates of each test are reported in Supplementary Table S1.

Exclusion criteria for CD patients included significant physical disability, pregnancy or breastfeeding, and any contraindications for MRI scanning, such as the presence of a pacemaker. Additionally, patients diagnosed with other inflammatory bowel diseases (IBD) aside from CD that could impact the study outcomes were also excluded.

For the healthy control (HC) group, participants were required to be healthy adults > 16 years of age, of any sex, with no history of IBD and able to provide informed consent. Individuals with a history of pre-existing gastrointestinal disorders that could influence study results, such as chronic liver disease, or abdominal cancer, were excluded.

### MRI protocol

All participants fasted for a minimum of 6 h before an MRI examination which was performed on a Philips 3 T Ingenia wide bore scanner (Philips, Best, The Netherlands) using a protocol which included additional standard anatomical and quantitative sequences not evaluated as part of this study. Participants were scanned supine in feet first orientation and a dStream anterior 16-channel coil was placed over the abdomen.

For this study, 2D ToF-MRA scans were acquired covering a FOV from the top of the hip joint to L3 vertebra. Pulse sequence parameters were: slice thickness, 3 mm; TE, 2.16 ms; TR, 9.8 ms; flip angle, 60°; FOV, 300 × 240 mm; matrix size, 336 × 336; resolution, 0.9 × 1.2 × 3 mm. The ToF-MRA scan was divided into five axial stacks, each consisting of 16 slices. Each stack was acquired during a breath-hold to avoid any respiratory motion artefacts in the images. Each breath-hold lasted 19 s with a resting period of 20 s between every breath-hold. The five stacks were combined to form a dataset of 80 slices covering the whole abdomen from the kidneys to the pelvic region.

2-point Dixon scans were acquired in the same anatomical region for secondary analysis of abdominal fat. The acquisition was performed using a 3D axial sequence with the following parameters: TR = 3.7 ms; TE = 1.19, 2.37 ms; flip angle = 10°; SENSE 2 acceleration, FOV = 300 × 240 × 400 mm; matrix size = 384 × 384 × 150; slice thickness = 6 mm (acquired) with reconstruction to 3 mm; and pixel spacing = 1.25 × 1.25 mm. The Dixon scan produced a stack of 150 slices covering the full abdominal volume, during three 10 s breath-hold scans (50 slices per breath hold).

### ToF-MRA quantification algorithm

Our study introduces a novel algorithm written using MATLAB (version R2020b by Mathworks, Natick, MA, USA) for quantifying mesenteric vascular branching in CD patients using ToF-MRA. The pipeline performs vessel enhancement, region-specific thresholding, rotating maximum-intensity projections (MIPs), skeletonisation, and branch-point detection to derive an Arborisation Index.

We choose branching points as the primary metric as they are topology-based and derived from skeletonised vessel graphs, making them less sensitive to absolute signal intensity, local calibre errors, or inter-scanner scaling than diameter or length measures. Junction counts provide a reproducible readout of network complexity that aligns with the mesenteric “comb sign,” an angiogenic remodelling pattern in active CD.

### Threshold selection method

A bespoke thresholding function was used because global methods (e.g., Otsu) [15] perform poorly on abdominal ToF-MRA. This is because the resulting image histogram is compact and skewed to low intensities, and peripheral subcutaneous fat (SAT) produces spurious high signal, so a single global threshold causes SAT signal leak.

To address this, ToF-MRA intensities were rescaled to [0,1]. Two in-plane zones were defined: a central intraperitoneal ROI (≈ the central third; includes viscera and VAT) and a peripheral band (predominantly SAT). A set of thresholds *(τ)* between 0.07 and 1.00 were evaluated. For each threshold, *τ*, the number of pixels with signal above the threshold was counted in the inner (*InnerCount(τ))* and outer (*OuterCount(τ))* zones, and a score was computed for each slice, balancing central vessel preservation against peripheral leak:

$$F\left( \tau \right) = InnerCount\left( \tau \right) - ~\frac{2}{3}OuterCount\left( \tau \right)$$

The factor of $\:\frac{2}{3}$ is to allow for the approximate difference in volume of the inner and outer zones. The threshold maximising this score ($\:{{\uptau\:}}^{*})\:$was found,

$$\:{\tau\:}^{*}={arg}\underset{\tau\:}{{max}}F\left(\tau\:\right),$$

and then a small fixed offset (Δ = 0.04) was added to allow for observed differences in the signal from visceral and subcutaneous fat, so that the final threshold was

$$\:\:\:{\tau\:}_{\mathrm{final}}={\tau\:}^{*}+\varDelta\:$$

This dual-zone criterion uses the SAT as a marker of VAT which would otherwise confound detection of vessels in the inner zone improving vessel extraction compared with traditional global thresholding.

After thresholding, the resulting image goes through several filtering operations to reduce noise, such as majority filtering, where each voxel is reassigned to the most common label within its surrounding 3 × 3 × 3 cube, hole filling to close internal gaps, and small-speckle removal using size-based connected-component filtering.

### Anatomical slab and masking

Analysis was confined to an axial abdominal slab from the superior margins of both femoral heads to the inferior endplate of L3. Manual masking was required only to exclude large non-mesenteric vessels arising from the liver and kidneys in 19 of 23 Crohn’s disease scans and in all healthy control scans. Masking was performed on the axial source images prior to definition of the analysed abdominal slab, and before determining whether renal structures were included within the region of interest. No other vessels or regions were manually edited, and masking was performed without reference to Arborisation Index values.

**Fig. 1:**
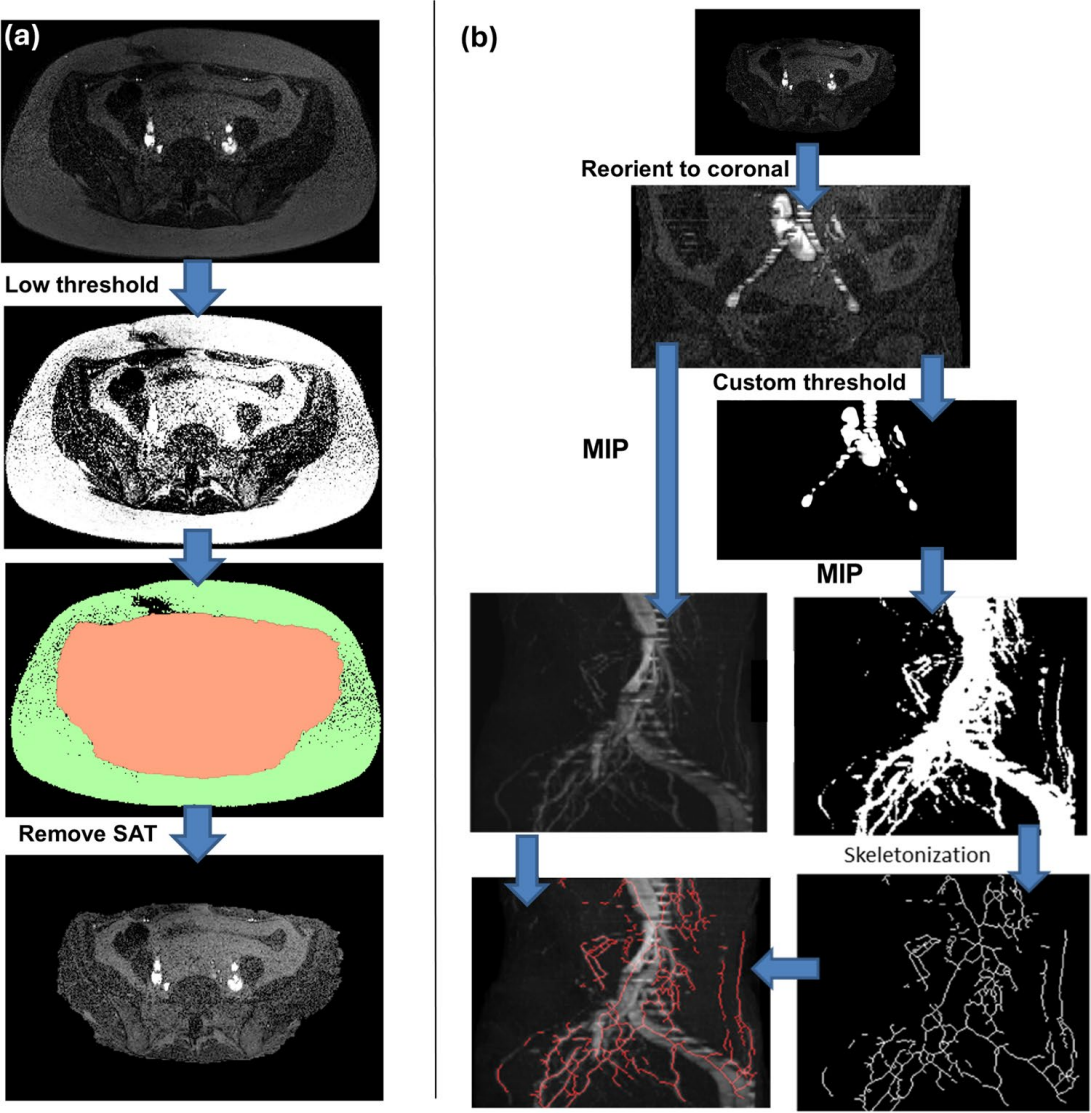
Workflow for abdominal fat segmentation (**a**) and mesenteric vessel analysis (**b**). Fat segmentation uses low-thresholding to remove SAT. Vessel analysis applies a custom threshold, MIP generation, and skeletonization to quantify branching

To restrict analysis to intraperitoneal vasculature, a low-intensity fat mask was created to highlight both visceral and subcutaneous adipose tissue; the subcutaneous compartment was then removed automatically, leaving an intraperitoneal mask for vessel analysis as shown in Fig. 1a. Analogous VAT/SAT separation is described under Abdominal fat analysis.

### Reorientation and rotating MIPs

The axial images are then resliced in the coronal plane before generating Maximum Intensity Projections (MIPs) at 20° rotational increments, resulting in 18 MIP images per scan. MIPs depict vessels along their full course, including segments that cross slice planes; the rotating views provide simple depth cues to assist in visualisation.

The MIP process is performed on both the original ToF-MRA (used for visual assessment and overlay of the final defined vessels) and the thresholded images (used for the quantitative analysis of vascular structures).

### Skeletonisation and branch detection

Thresholded MIPs were skeletonised to 1-pixel-wide centrelines using topology-preserving morphological thinning [16]. Isolated and short terminal spurs (≤ 3 pixels) were pruned to reduce noise. To prevent horizontal banding artefacts from being misidentified as vessels, horizontal line segments ≥ 7 pixels were detected and removed with a row-wise size filter before branch detection. Branch points were identified by scanning each skeleton pixel’s 8-neighbourhood and counting the number of connected pixels; pixels with ≥ 3 skeleton neighbours were labelled as branch points. Crossings were counted as branch points. The procedure was applied identically to all 18 MIPs (Fig. 1b).

### Arborisation index

The final output includes a rotated series of MIPs of the ToF-MRA scan, with vessels highlighted. The key metric generated is the Arborisation Index, calculated as the sum total number of branching points found all the MIP image divided by the number of MIPs.

### Abdominal fat analysis

To assess the relationship between vascularization and visceral fat, two-point modified Dixon acquisitions were used to produce water-only and fat-only volumes. An in-house, automated MATLAB^®^ segmentation (reported previously [17–20]) was used to quantify the volumes of subcutaneous and visceral fat from these scans. For comparability with the ToF-MRA analysis, the same axial slice range (femoral heads to L3) was selected manually on the Dixon series, and VAT and SAT volumes were reported within this slab.

### Statistical analysis

Statistical analysis was performed in Microsoft Excel (2023) and Pingouin (Python). Normality was assessed with the Shapiro–Wilk test. Between-group differences were tested with unpaired, two-tailed t-tests (*p* ≤ 0.05). Results are reported as mean ± SD. Effect sizes were expressed as Cohen’s d (small 0.2, medium 0.5, large ≥ 0.8) [21].

For repeatability, a second ToF-MRA was acquired at the end of the session in a subset of participants (CD *n* = 14, HC *n* = 5). For each participant, CoV% = SD(repeats) × 100/mean(repeats) was calculated for the arborisation index; participant CoVs were averaged within each group. Repeatability was categorised as poor (> 30%), acceptable (20–30%), good (10–20%), or excellent (≤ 10%) [22, 23].

Inter-subject variation was estimated from CoV% across participants within each group using the first measurement only: CoV% = SD(across participants) × 100/mean(across participants) which was compared with a cross-participant variation.

BMI-matched analysis (1:1, ± 1 kg/m²) paired CD with HC; paired t-tests compared Arborisation Index within pairs. The Index was also compared between CD patients with and without prior bowel resection.

Linear regression and Pearson’s correlation coefficient assessed relationships among Arborisation Index, BMI and visceral adipose tissue volume. CRP values below the assay limit of detection were set to LoD/2.

## Results

### Participant demographics

**Fig. 2:**
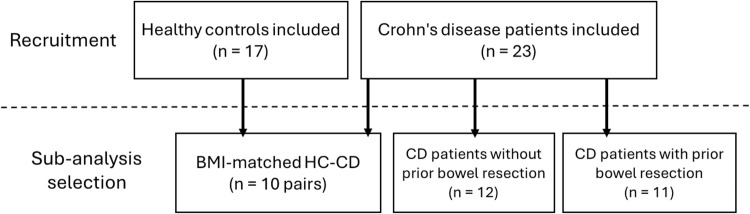
Study flow diagram showing included healthy controls and Crohn’s disease patients, BMI-matched HC–CD pairs, and stratification by prior bowel resection

A total of 23 CD patients (15 males, 30 ± 10 years, BMI 25 ± 4 kg/m^2^) and 17 HCs (8 males, age 44 ± 14 years, BMI 29 ± 5 kg/m^2^) were recruited for this study between 2021 and 2023. For CD patients, average CRP was 11.0 ± 16.8 mg/L (*n* = 19) and faecal calprotectin 744 ± 1168 µg/g (*n* = 11) and global sMaRIA score of 4.4 ± 3.7(*n* = 21). Eleven CD patients (48%) had a prior CD-related intestinal resection. Full demographic and clinical details are provided in Supplementary Tables S1 (CD) and S2 (HCs). Shapiro–Wilk tests showed no evidence of non-normality for the Arborisation Index (CD: W = 0.97, *p* = 0.69; HC: W = 0.94, *p* = 0.34). Participant inclusion and subgroup selection are summarised in Fig. 2.

### Arborisation index: Crohn’s disease vs. healthy controls

**Fig. 3:**
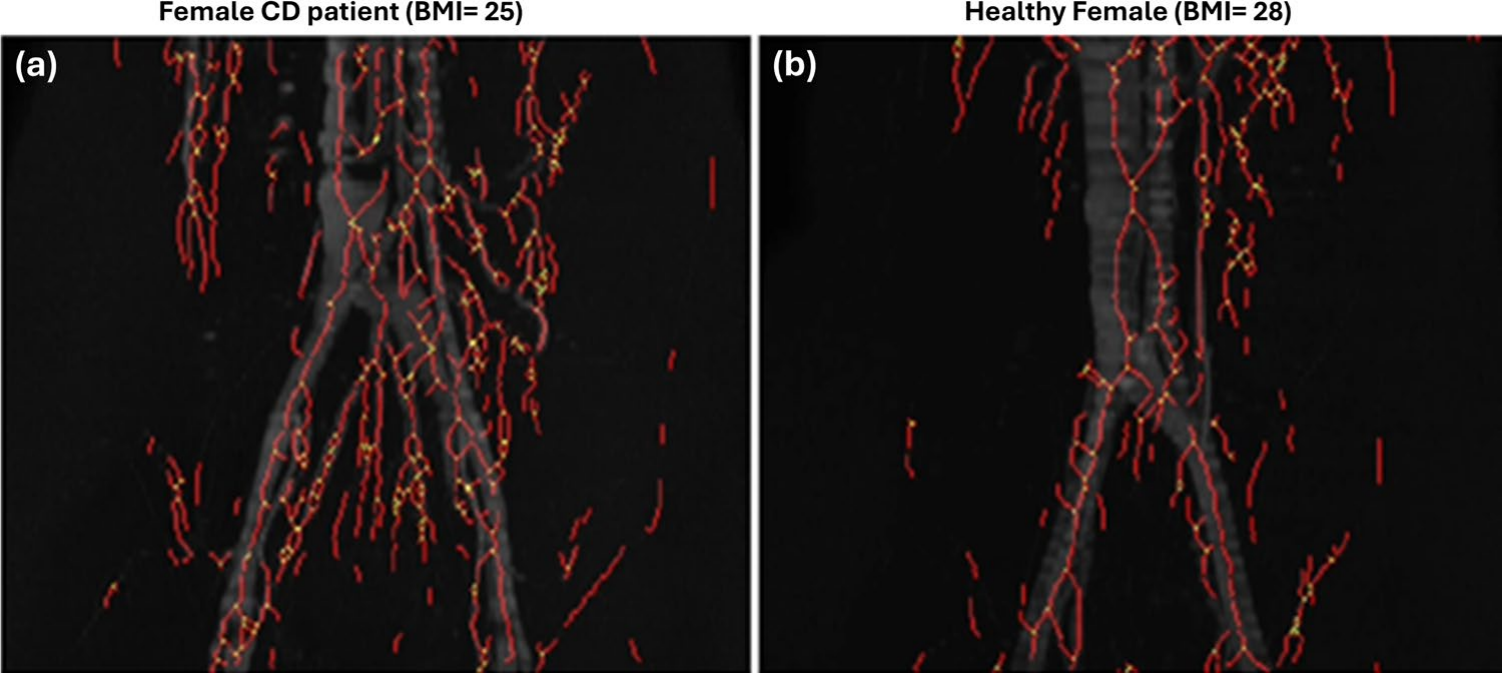
Examples of ToF-MRA MIPs of a female CD patient (**a**) and a female healthy control (**b**), with traced vessels in red and branching points in yellow

**Fig. 4:**
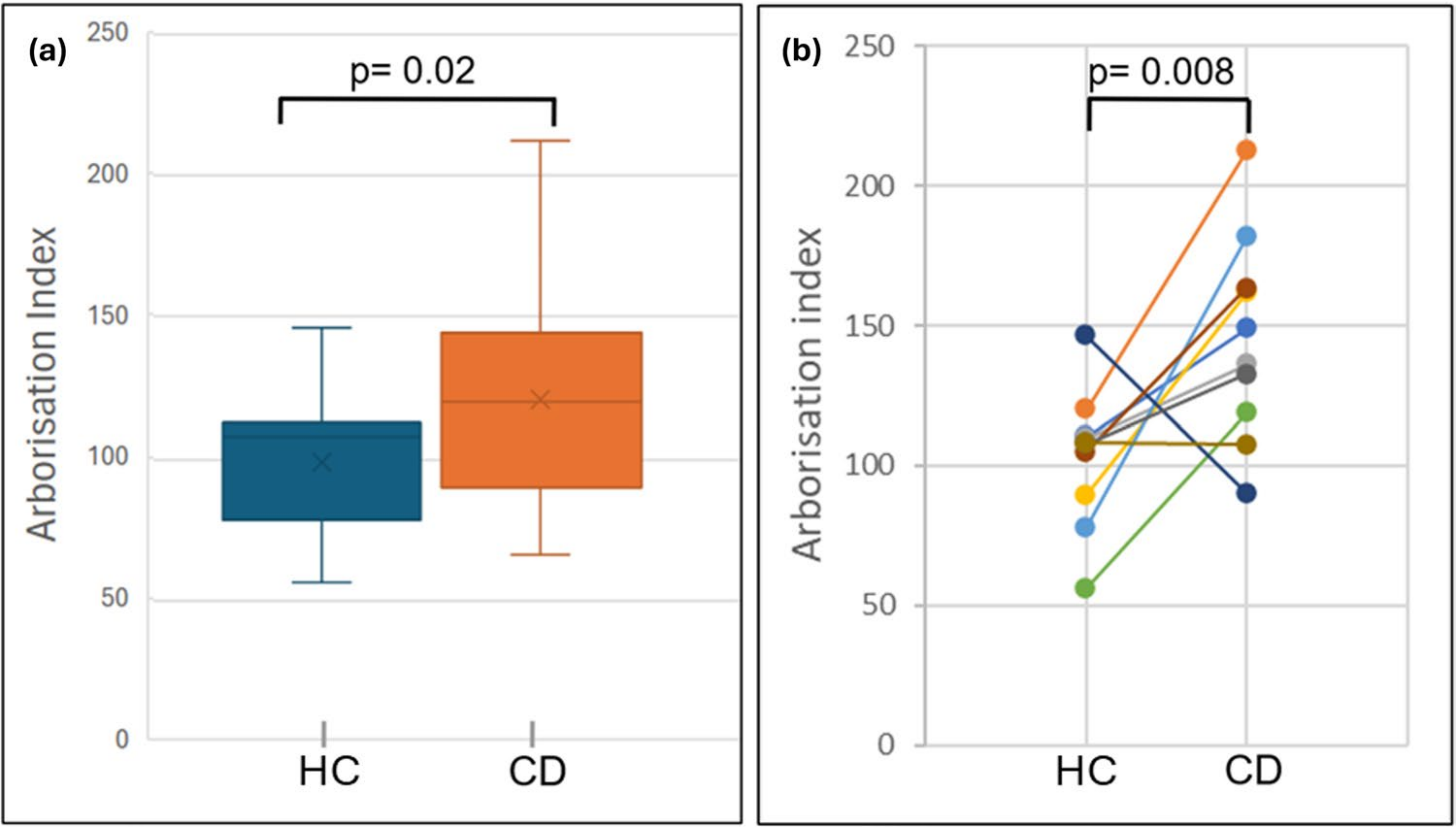
**a** Box-and-whisker plot of the Arborisation Index in healthy controls HCs and CD patients. The centre line indicates the median, boxes represent the interquartile range (IQR), whiskers denote the minimum and maximum values, and crosses indicate the mean. **b** paired dot plot comparing the Arborisation Index of 10 HCs and 10 CD patients matched for BMI

CD patients showed a significantly higher Arborisation Index when compared with HCs (mean Arborisation Index in HCs = 98.4 ± 24.6 and CD = 122.7 ± 37.1; *p* = 0.02, d = 0.7), see Figs. 3 and 4.

In a planned first sub-analyses for 10 CD patients and 10 HCs matched for BMI, the CD group still showed a significantly higher Arborisation Index when compared to the HC cohort (HCs = 103 ± 24.3 vs. 145.3 ± 36.3; *p* = 0.008, d = 1.4), (Fig. 4B) suggesting that the difference may be due to vasodilation secondary to disease activity rather than BMI variations.

Arborisation Index values were similar in CD patients with a prior bowel resection (116.1 ± 30.6, *n* = 11) and those without (128.7 ± 42.6, *n* = 12; *p* = 0.42). This suggests that historical surgical intervention did not materially influence the mesenteric vascular pattern captured by ToF-MRA in this cohort.

### Biomarker technical validation and variability

Inter-subject variation (across-participant CoV) was 30% in CD and 25% in HCs. Intra-subject variability, from 19 pairs of repeated ToF-MRA scans (CD *n* = 14; HC *n* = 5), yielded mean CoV of 8.9 ± 6% for CD patients and 8.5 ± 5% for HCs, indicating repeatability markedly better than Inter-subject variation.

**Fig. 5:**
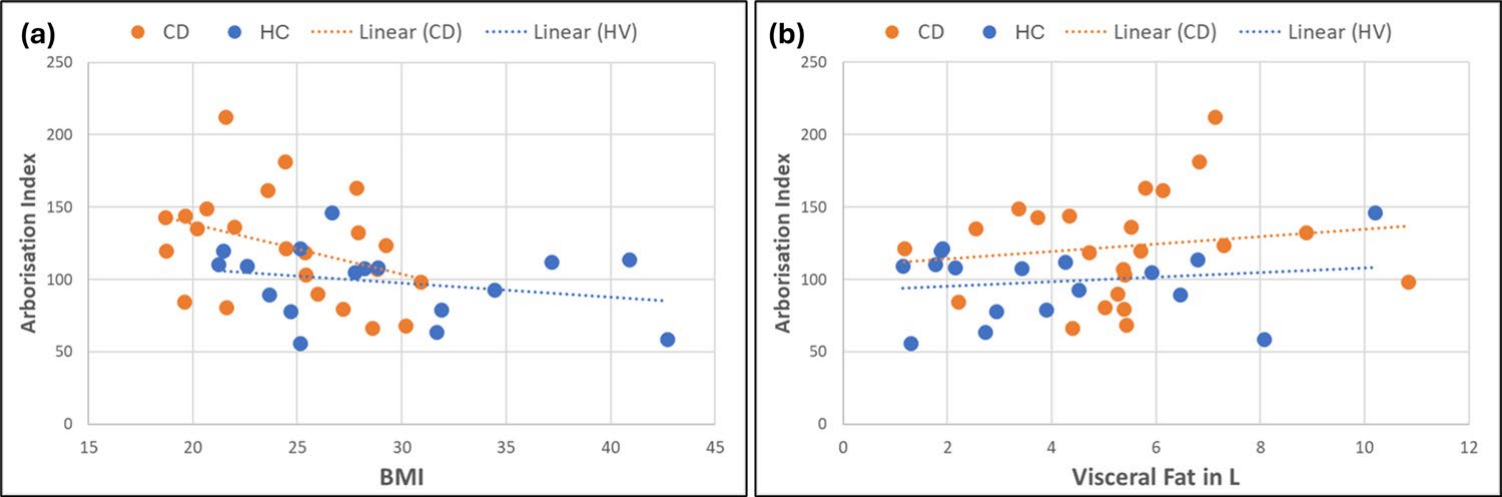
Arborisation Index plotted against BMI (**a**) and visceral fat volume (**b**) in CD patients and healthy controls

No significant correlation was observed between the Arborisation Index and BMI (HCs: *r* = −0.26, *p* = 0.31; CD: *r* = −0.36, *p* = 0.09). Similarly, the Arborisation Index demonstrated no significant correlation with visceral fat volumes measured from 2-point Dixon MRI scans (CD: *r* = 0.145, *p* = 0.510; HC: *r* = 0.171, *p* = 0.512), as shown in Fig. 5, further suggesting that the Arborisation Index is independent of visceral fat growth.

## Discussion

In this study, we presented a new metric, the Arborisation Index, for assessing changes in the abdominal vasculature in CD, and described a unique MRI ToF-MRA protocol and automated quantification algorithm for measuring this. The study findings showed that the Arborisation Index is significantly higher in CD patients compared to HCs and this difference persisted even after matching for BMI. The absence of correlation with BMI or visceral adipose tissue suggests that the Arborisation Index is not trivially driven by body composition or fat-related signal effects. However, given the exploratory design and limited cohort size, the present findings should be interpreted as demonstrating technical feasibility and measurement stability rather than a definitive pathophysiologic attribution.

Most automated or semi-automated assessments of arterial vasculature —size, symmetry, branching characteristics, and bifurcation angles— have been developed for use in brain imaging [24, 25]. However, translation of these methods into abdominal imaging is not trivial due to motion, larger imaging volume, abdominal fat signal interference and lower image resolution (due to the need to acquire a scan during a breath-hold).

Vessels branching-based metrics offer a quantitative analogue of the mesenteric “comb sign” because branch-point density scales with angiogenic network complexity and has long been used as a validated readout in vascular image analysis after segmentation and skeletonization [26, 27]. In this cohort, test–retest repeatability was good (within-subject coefficient of variation, CoV: 8.9 ± 6% in CD; 8.5 ± 5% in HCs), indicating low intra-subject variation and supporting branch-point counts as a stable imaging-derived metric for Mesenteric hyperaemia.

While an association between mesenteric hypervascularity and CD has been established in the literature, CD studies use a subjective assessment by radiologists on CTE images as an indicator for CD activity [5, 28]. Wu et al. quantified the comb sign retrospectively by placing twenty 1 cm² ROIs on CTE and manually counting vessels, averaging counts per subject. They analysed 72 CD patients and 42 controls, subdividing CD into active vs. inactive based on Rutgeerts’ score. Their results show that quantitative comb-sign values were higher in CD than controls and higher in active than inactive CD. Reported accuracy for predicting CD activity was 78.4% (arterial) and 80.0% (venous) [6].

MRI can be used to visualise the comb sign on post-contrast T1-weighted MRI [29, 30], but MRI based CD activity indices such as Clermont [31] and sMaRIA [14] scores do not explicitly include assessment of the mesenteric ‘comb sign’ among their defined components, probably due to the difficulty in objectively quantifying hypervascularity in contrast free MRI. ToF-MRA overcomes this limitation by exploiting flow-related signal enhancement to visualise the vasculature, with increased conspicuity in active disease due to the hyperaemia and vasodilatation characteristic of intestinal inflammation. The lack of effect of prior bowel resection on the Arborisation Index indicates that this metric reflects current haemodynamics rather than fixed anatomy, although this finding should be interpreted cautiously given the small sample size.

Responsiveness of mesenteric blood flow hemodynamics to anti-TNF therapy has been reported in both adults and children. In adults, anti-TNF therapy was associated with early improvement in superior mesenteric artery blood flow [32]. In paediatric CD, MRI-based measurements demonstrated a significant reduction in superior mesenteric artery and vein blood flow by 6 weeks after treatment initiation, with further decreases at 6 months [33]. Longitudinal studies, involving imaging at multiple time points and with a larger sample size, should be undertaken to address the value of the responsiveness of Arborisation Index.

Abdominal ToF-MRA is inherently sensitive to intraluminal signal within the bowel, which can appear hyperintense and confound vessel detection. Participants therefore fasted for at least 6 h prior to imaging to reduce intraluminal content and associated non-vascular signal. While fasting may also reduce small bowel motility, residual peristalsis and luminal fluid can still generate hyperintense signal unrelated to mesenteric perfusion. In addition, fasting requirements may complicate integration into routine clinical workflows, where MRE protocols typically involve oral bowel preparation. Several filtering and noise-correction steps were therefore implemented, including region-specific thresholding, and skeleton-based pruning, to minimise background signal and misregistration of branching points. Despite these measures, the arborised vascular projections occasionally include short terminal branches that likely reflect residual segmentation artefacts or partial-volume effects. Additionally, scans required 19-second breath-holds, which some patients could find difficult. In 2D time-of-flight MRA, signal intensity is dominated by inflow-related enhancement, which preferentially highlights faster inflowing arterial blood, while venous signal is relatively attenuated due to slower flow and saturation effects. Nevertheless, venous structures with sufficient through-plane flow may still contribute to the observed signal and cannot be completely excluded. All imaging was performed on a single 3 T scanner, which provides increased sensitivity for ToF-based methods. However, 1.5T systems are more widely used for routine abdominal imaging, and inter-scanner and inter-vendor variability were not assessed in this study. Given the known sensitivity of ToF-MRA to field strength and sequence implementation, future work should evaluate the robustness of the Arborisation Index across field strengths and vendors.

Inter-subject variation of the Arborisation Index (CoVs of 25% for HCs and 30% for CD patients) was relatively high, which may be partly attributable to the small sample size of the study. Although the vessel segmentation pipeline is otherwise automated, exclusion of hepatic and renal vessels required limited manual masking, which reduces full end-to-end automation and represents a practical limitation of the current implementation.

Confounding factors may have influenced the observed differences between the groups. The CD patients were older than the HCs and age could potentially impact mesenteric vessels’ morphology and could therefore affect the Arborisation Index. Similarly, other demographic factors such as height and gender may also have influenced the results. Further validation to standard disease activity measures is needed across larger cohorts to better investigate the utility and reversibility of this biomarker as a non-invasive measure of disease activity.

In summary, this study demonstrates the technical feasibility and repeatability of automated mesenteric vascular quantification using non-contrast ToF-MRA in Crohn’s disease. While group-level differences were observed, the Arborisation Index should currently be regarded as a reproducible imaging-derived metric rather than a validated biomarker of disease activity. Larger, longitudinal, and multicentre studies are required to establish biological meaning, responsiveness to treatment, and clinical utility.

## Supplementary Information

Below is the link to the electronic supplementary material.

Supplementary Material 1

Supplementary Material 2

## Data Availability

The datasets used and/or analysed during the current study are available from the corresponding author upon reasonable request.

## Abbreviations

Anti-TNF: Anti-Tumor Necrosis Factor
BMI: Body Mass Index
CD: Crohn’s Disease
CRP: C-reactive Protein
CT: Computed Tomography
CTE: Computed Tomography Enterography
CoV: Coefficient of Variation
ESR: Erythrocyte Sedimentation Rate
FCP: Faecal Calprotectin
FOV: Field of View
HC: Healthy Control
IBD: Inflammatory Bowel Disease
L3: Third Lumbar Vertebra
LoD: Limit of Detection
MIP: Maximum Intensity Projection
MRA: Magnetic Resonance Angiography
MRE: Magnetic Resonance Enterography
PCC: Pearson’s Correlation Coefficient
PC-MRA: Phase-Contrast Magnetic Resonance Angiography
ROIs: Regions of Interest
SAT: Subcutaneous Adipose Tissue
SD: Standard Deviation
sMaRIA: Simplified Magnetic Resonance Index of Activity
TE: Echo Time
ToF: Time-of-Flight
TR: Repetition Time
VAT: Visceral Adipose Tissue
