# Supplementary material for: Automated quantification of mesenteric hyperaemia in Crohn’s disease using time-of-flight magnetic resonance angiography
Source: Abdom Radiol (NY). 2026 Feb 6;51(8):3748–57. doi: 10.1007/s00261-026-05384-6 (PMC13388376; doi:10.1007/s00261-026-05384-6)
Supplement: Supplementary file 1 — Supplementary Material 1 [file 261_2026_5384_MOESM1_ESM.docx]

Supplementary Table S1: Demographic and clinical characteristics of Crohn’s disease patients, including biomarker data, MRI dates, sMaRIA scores, and surgical history.

| no. | Age | Sex | BMI | CRP (mg/L) | CRP– Study MRI interval (days) | FCP (ug/g) | FCP– Study MRI interval (days) | sMaRIA scores | Clinical MRI–Study MRI interval (months) | Arb index | Surgical history (timing relative to study MRI, years) |
| --- | --- | --- | --- | --- | --- | --- | --- | --- | --- | --- | --- |
| 1 | 45 | F | 25.4 | <5 | –133 | 52 | –108 | NA | NA | 118.8 | Pan proctocolectomy (~18 yrs); refashioning of ileostomy (~10 yrs); small bowel resection (~6 yrs) |
| 2 | 27 | M | 20.2 | 4 | +107 | 2702 | –123 | 2 | –2.5 | 135.3 | Small bowel resection (~10 yrs); further small bowel resection (~9 yrs); stricturoplasty (~8 yrs) |
| 3 | 41 | M | 26 | N/A | NA | 57 | –288 | 2 | –2.4 | 90.2 | No prior bowel surgery |
| 4 | 56 | M | 23.6 | 2 | –5 | N/A | NA | 3 | –21.9 | 161.7 | No prior bowel surgery |
| 5 | 68 | F | 19.6 | 2 | +17 | N/A | NA | 14 | –1.5 | 144.2 | Ileocolic resection and re-anastomosis (~0.5 yrs) |
| 6 | 50 | F | 20.7 | <1 | +76 | 182 | –206 | 2 | –1.0 | 148.9 | Colectomy with ileostomy and retained rectal stump (~19 yrs) |
| 7 | 54 | M | 24.4 | 3 | –6 | 62 | –118 | 1 | +0.4 | 181.7 | No prior bowel surgery |
| 8 | 50 | M | 29.2 | 19 | –94 | N/A | NA | N/A | NA | 123.5 | Right hemicolectomy (~26 yrs); ileocolic resection (~25 yrs); further ileocolic resection (~7 yrs) |
| 9 | 49 | F | 27.8 | 6 | –56 | N/A | NA | 13 | –19.2 | 163.3 | Chronic bowel resection and defunctioning ileostomy (~6 yrs); perianal seton insertion (~6 yrs); ileostomy closure (~2 yrs); abscess incision for enterocutaneous fistula (~2 yrs) |
| 10 | 61 | M | 28.8 | N/A | NA | 34 | –11 | 2 | –5.3 | 107.3 | Ileocecectomy (~12 yrs) |
| 11 | 36 | F | 27.9 | N/A | NA | N/A | NA | 1 | –10.8 | 132.4 | No prior bowel surgery |
| 12 | 25 | F | 24.4 | N/A | NA | N/A | NA | 4 | –2.4 | 121.4 | No prior bowel surgery |
| 13 | 57 | M | 21.6 | 6 | –134 | 252 | –73 | 4 | –6.1 | 212.6 | No prior bowel surgery |
| 14 | 34 | F | 19.6 | 53 | +20 | N/A | NA | 7 | –9.8 | 84.7 | Total colectomy with end ileostomy (~12 yrs); ileorectal anastomosis (~8 yrs); perianal fistulotomy (~2 yrs); laparotomy (~2 yrs); small bowel resection with end ileostomy (~2 yrs); percutaneous drainage of iliopsoas sepsis (~1 yr); small bowel resection with fistula repair and double-barrel jejunostomy (~1 yr) |
| 15 | 81 | M | 22 | 2 | –274 | N/A | NA | 2 | –11.4 | 136.2 | No prior bowel surgery |
| 16 | 52 | M | 18.7 | 8 | +23 | 499 | +106 | 5 | –36.6 | 143.1 | No prior bowel surgery |
| 17 | 33 | M | 18.7 | 40 | –17 | 939 | +92 | 4 | +0.6 | 120.1 | No prior bowel surgery |
| 18 | 48 | F | 30.9 | 4 | –9 | 58 | –162 | 2 | –2.1 | 98.2 | No prior bowel surgery |
| 19 | 24 | M | 21.6 | 49 | –5 | N/A | NA | 9 | –0.8 | 80.6 | No prior bowel surgery |
| 20 | 40 | M | 28.6 | 5 | +209 | N/A | NA | 3 | +3.3 | 66.4 | No prior bowel surgery |
| 21 | 37 | M | 27.2 | 1 | –26 | N/A | NA | 2 | –4.2 | 79.8 | Terminal ileal resection (~2 yrs) |
| 22 | 38 | M | 25.4 | 1 | –19 | N/A | NA | 2 | –5.6 | 103.1 | Right hemicolectomy with ileostomy formation (~21 yrs) |
| 23 | 28 | M | 30.2 | 1 | –35 | 3347 | –179 | 8 | –5.4 | 68.4 | Small bowel resection with primary anastomosis and enterocutaneous fistula formation (~2 yrs) |

BMI=Body mass index; CRP=C-reactive protein; FCP= Faecal calprotectin; Arb= Arborisation.
