# Supplementary material for: Automated quantification of mesenteric hyperaemia in Crohn’s disease using time-of-flight magnetic resonance angiography
Source: Abdom Radiol (NY). 2026 Feb 6;51(8):3748–57. doi: 10.1007/s00261-026-05384-6 (PMC13388376; doi:10.1007/s00261-026-05384-6)
Supplement: Supplementary file 2 — Supplementary Material 2 [file 261_2026_5384_MOESM2_ESM.docx]

Supplementary Table S2. Demographic characteristics and arborisation index values of healthy controls.

| no. | Age | Gender | BMI | Arb index |
| --- | --- | --- | --- | --- |
| 1 | 25 | F | 34.44961 | 92.94 |
| 2 | 29 | M | 23.63769 | 89.3 |
| 3 | 22 | F | 28.21869 | 107.8 |
| 4 | 24 | M | 31.89981 | 79.4 |
| 5 | 34 | M | 24.6649 | 78 |
| 6 | 29 | F | 25.11617 | 121.7 |
| 7 | 31 | F | 40.88889 | 113.8 |
| 8 | 24 | F | 21.2071 | 110.4 |
| 9 | 28 | M | 31.67347 | 63.7 |
| 10 | 27 | F | 21.45329 | 119.9 |
| 11 | 24 | F | 22.58271 | 109.5 |
| 12 | 46 | M | 27.7551 | 105 |
| 13 | 32 | F | 28.84728 | 108.2 |
| 14 | 26 | M | 25.12783 | 56.1 |
| 15 | 23 | M | 42.70417 | 59 |
| 16 | 24 | F | 37.18025 | 112.3 |
| 17 | 61.5 | M | 26.6436 | 146.2 |

BMI=Body mass index; CRP=C-reactive protein; FCP= Faecal calprotectin; Arb= Arborisation.
